# The Influence of Raster Angle and Moisture Content on the Mechanical Properties of PLA Parts Produced by Fused Deposition Modeling

**DOI:** 10.3390/polym13020237

**Published:** 2021-01-12

**Authors:** Mohammed Algarni

**Affiliations:** Mechanical Engineering Department, Faculty of Engineering, King Abdulaziz University, P.O. Box 344, Rabigh 21911, Saudi Arabia; malgarni1@kau.edu.sa

**Keywords:** fused deposition modeling, PLA, raster angle, moisture content, mechanical properties, process parameters

## Abstract

The additive manufacturing (AM) processes and technologies of 3D-printed materials and components using fused deposition modeling (FDM) are currently very popular and widely used for building parts and prototypes. Many manufacturing parameters can affect the strength and strain of the manufactured parts. The manufacturing parameters may be altered to reach an optimum setting for highly effective parts or components. This research studies the influence of the raster angle and the moisture content percentages on the mechanical properties of 3D printed polylactic acid (PLA) material. The three raster angles tested in this research were 0°, 45°, and 90°. The moisture content of the PLA material was altered to verify its effect on the mechanical properties. Twenty-seven specimens were subjected to tensile tests to examine the effect of different manufacturing parameters. The results show the specimens with a 90° raster angle and 10% moisture content have the optimum strength and strain mechanical properties.

## 1. Introduction

In recent years, additive manufacturing (AM) technologies have been used for 3D rapid fabrication that involves different manufacturing disciplines, such as computer-aided design (CAD) and information and material science. 3D printing applications have been expanding rapidly due to the minimal cost of the production process, low supervision and maintenance cost, and simple material change [1]. Different 3D printing processes have been developed using numerous materials and methods [2,3,4,5,6,7,8,9,10,11,12,13,14,15]. One of the most common 3D printing technologies is fused deposition modeling (FDM), which is a layer-by-layer technique using CAD and computer-aided manufacturing (CAM). The main advantages of FDM are the ease of manufacturing complex shapes (i.e., parts with hollow cavities and parts within parts) and the ability to include various materials into a single part (i.e., materials with different colors and mechanical properties). However, the disadvantages of FDM are the limitations in the properties of the available materials [16,17]. Additionally, the strength of the 3D-printed parts made by FDM is lower than the strength of parts made by the same material with a conventional process such as injection molding [18,19].

Polylactic acid (PLA) is an eco-friendly material made of renewable resources, making it biocompatible and recyclable. It is approved by the US Food and Drug Administration (FDA) [20]. PLA has been extensively researched and has become a promising candidate material to replace traditional petrochemical-based polymers in different medical applications (implants) and food packaging due to its non-toxic origin (sugar cane and starch). 

FDM is a manufacturing process that is affected by many parameters that may alter the quality and mechanical properties of the part. Many researchers have investigated multiple parameters to improve the performance of the 3D-printed parts and components [5,6,7,8,9,10,11,12,13,14,15]. Chacón et al. [21] investigated how the strength and the ductility of PLA specimens are affected by the layer thickness. The number of layers required to print a specimen increases as the layer thickness decreases. Thus, the manufacturing time and cost increase. They concluded that increasing the layer thickness results in increasing the tensile strength and decreasing the ductility. This can be explained by specimens with increased layer thickness requiring fewer layers and, therefore, fewer layer bonds. Layer bonds decrease the specimens’ strength. Research by Ahn et al. [22] explored the effect of multiple parameters on 3D parts printed from acrylonitrile butadiene styrene (ABS). The parameters were bead width, air gap, model temperature, and raster angle (RA). The study concluded that the raster angle and the air gap affect the specimen’s strength, while the model temperature and bead width have a low effect on the strength. Moreover, Hibbert [23] studied the effect of the interior fill, layer thickness, and raster angle on the quasi-static response of ABS 3D-printed parts. The results show that the layer thickness and the interior fill altered the strength, whereas the raster angle influenced the failure mode. A study by Durgan et al. [24] showed that the build orientation parameter (vertical, horizontal, and perpendicular), known as the specimen position, has a greater effect than the raster angle parameter on the mechanical properties. A recent study by Zhang et al. [25] investigated the effect of the raster angle on Al/PLA composite parts. The results show that the raster angle affects the strength, fracture morphology, strain, and failure mode. Amza et al. [26] studied the inclusion of ultra-high-molecular-weight polyethylene fibers in PLA without major degradation during the melting process. The study concludes that the quality of the fabrication depends on the fiber’s orientation with respect to extrusion pathing. 

Natural fibers have almost 6 to 10% moisture content (MC) by weight. The presence of moisture in natural fibers creates voids and bulges in the fiber’s composition. A study by Zaldivar et al. [27] evaluated the performance of several dogbone shape specimens made of ULTEM 9085 filament with different moisture content. The study showed a significant decrease in the strain at fracture and tensile strength as the moisture content increase. Van den Oever et al. [28] found that a moisture content of 0.3 wt% (±0.1%) has minimal influence on the mechanical properties of a printed part. Furthermore, a study by Fujiura et al. [29] showed that mechanical properties decreased due to hydrolysis. The research group revealed the importance of dehydrating PLA to improve the mechanical performance of the PLA specimens. Kariz et al. [30] examined the effect of moisture content on the wood powder and PLA mixture. The study concludes that the modulus of elasticity weakens as the moisture content increases. A study by Halidi et al. [31] showed the effect of moisture content in ABS on the nozzle blockage on the liquefier in a fused deposition modeling (FDM) machine. The nozzle blockage causes physical, morphological, and thermal stability changes to the ABS specimens. Another study by Kim et al. [32] investigated the mechanical properties of FDM-modeled specimens under different moisture and temperature conditions. The results show that specimens built in an environment with higher moisture content have 70% lower strength than specimens built in a dry environment.

In this research, the mechanical properties of PLA 3D-printed specimens were studied. Tensile tests were conducted for PLA specimens built with different raster angles and moisture content. The effect of the raster angle and the moisture content process parameters on the mechanical properties of PLA specimens was examined. Finally, the optimum raster angle and moisture content are identified and presented.

## 2. Experimental Procedure

### 2.1. Experimental Material

The properties of the PLA filament used in this research are shown in Table 1. This filament is manufactured by eSUN® (eSUN, Shenzhen, China). The PLA filament is hard yet flexible, and it is the most used material in FDM systems to produce quick products, hard parts, and reliable prototypes. PLA is made with eco-friendly materials and renewable resources that demand low energy (compared to other petrochemical-based materials) for processing. Thus, PLA is used to make many products that are used every day, such as beauty prints, kids’ toys and prototypes, and low-stress applications. The material used was initially characterized by the manufacturer, as in Table 1, which is used later to determine the effect of the raster angle and moisture content.

### 2.2. Specimens Design

**Figure 1:**
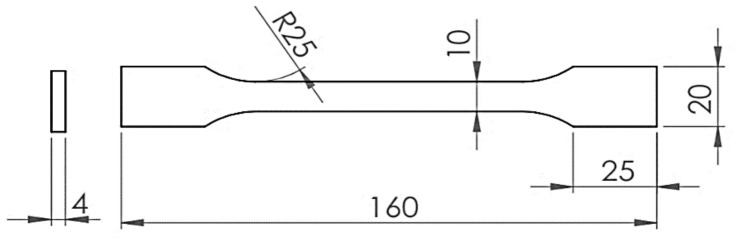
Dogbone dimensions in mm.

The tensile specimens were designed, prepared, and printed as a flat dogbone shape according to the American Society for Testing and Materials (ASTM) standard. The shapes were tested according to the Test Methods for Tensile Properties of Plastics (ASTM D638) [34], which is widely used for plastic specimens. The specimens are 4 mm thick, and the gauge length is 85 mm. Other geometries and dimensions are illustrated in Figure 1.

### 2.3. Design of Experiment and Printing Variables

The FDM printer used to fabricate the PLA specimens is a commercial Ultimaker 2 + TM (Ultimaker B.V., Utrecht, The Netherlands) 3D printer known for using a heated glass plate (build plate) with a swappable nozzle and an open filament roll system. The filament roll and nozzle diameter are 2.85 mm and 0.4 mm, respectively. The FDM machine functions at ambient room temperature and has a 180 to 260 °C nozzle temperature. Ultimaker Cura 4.6.2 software integrated with solid modeling CAD SolidWorks program by Dassault Systèmes (Vélizy-Villacoublay, France) is used to operate the 3D printer. The PLA was extruded and melted at the nozzle, then deposited on the build plate in multiple layers following a specific horizontal route to create the specimens. The layers harden and hold together while cooling as a single mass. Voids and defects were avoided during the manufacturing process by setting the thickness of the shell equal to the nozzle diameter. The specimens were built flat (horizontally) on the build plate. 

**Figure 2:**
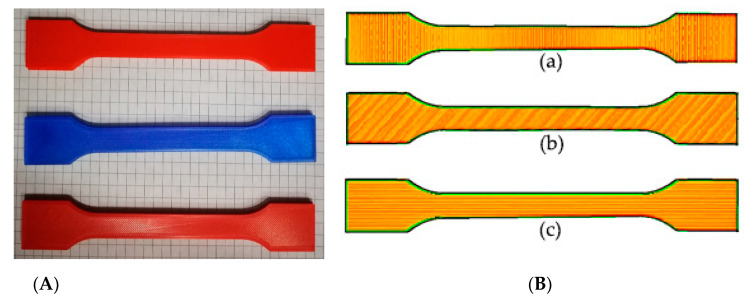
Printed specimens from top to bottom 0°, 45°, 90°, respectively (**A**). Raster angle (infill orientation) of tests specimens: (a) 0°, (b) 45°, (c) 90° (**B**).

**Figure 3:**
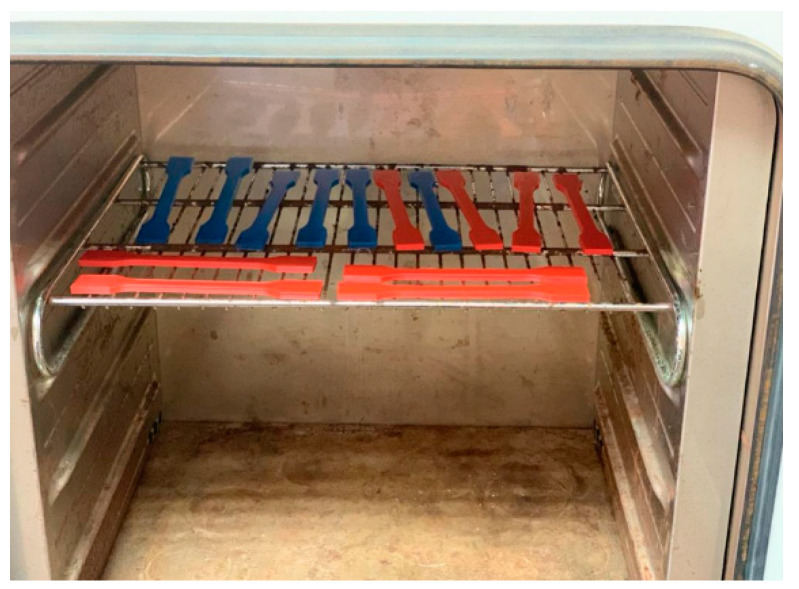
Specimen setup in the dryer.

Two design of experiment (DOE) variables are examined in this research: (A) raster angle (RA) and (B) moisture content (MC). The specimens are printed with three raster angles: 0°, 45°, 90°, as shown in Figure 2. The raster angle is defined by the infill orientation or the printing direction of the filament during the FDM process (Figure 2) where 0° is perpendicular to the loading direction and 90° is parallel to the loading direction. The moisture content was altered to 1%, 5%, and 10%. The specimens with 1% moisture content were dehumidified in an electric coiled dryer (BINDER INC., Tuttlingen, Germany) in Figure 3 at 45 °C for 5 h. The specimens with 5% and 10% moisture content were dehumidified at the same temperature for 3 and 1 h, respectively.

The DOE factors are the two variable printing processes: raster angle and moisture content. Table 2 shows these factors with the corresponding levels. Table 3 is the experimental design matrix that shows nine experiments based on the number of levels of each corresponding factor. The specimens with similar raster angle were printed using the same filament. Furthermore, other printing parameters employed in the study are fixed using the recommended standard profile, as shown in Table 4.

### 2.4. Mechanical Testing Setup

**Figure 4:**
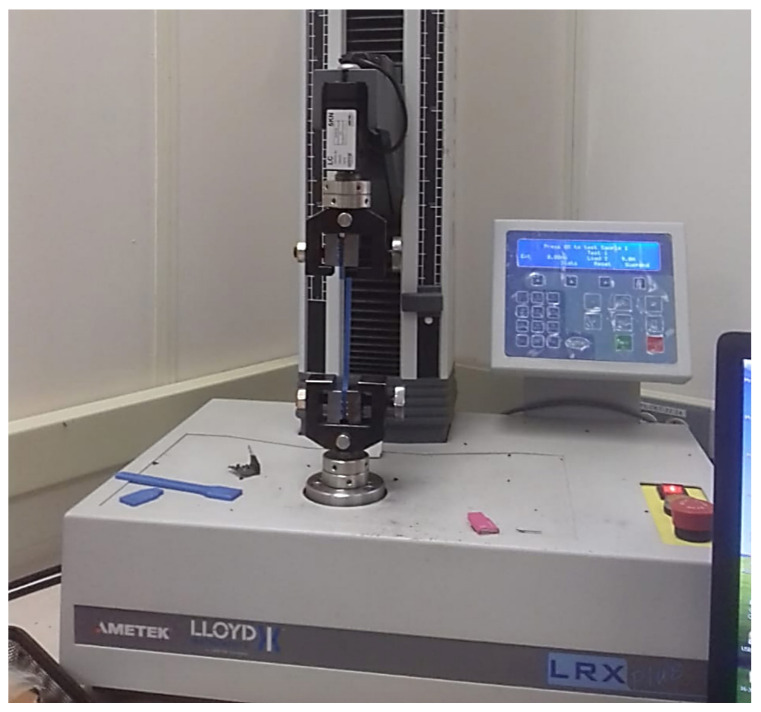
The setup of the AMETEK load frame with 5 kN load capacity.

The monotonic tensile tests were performed in accordance with ASTM D638 [34] using an AMETEK load frame (AMETEK STC, Berwyn, Pa, US) with a 5 kN load cell capacity and at 5 mm/min crosshead speed (Figure 4). The tensile tests results were converted from force (N) vs. elongation (%) into stress (MPa) vs. strain (mm/mm) curves. Each level test had three specimens, and the tensile properties were analyzed and averaged to investigate the role of the raster angle and moisture content on the mechanical properties. The study identified the change in the ultimate tensile strength (UTS), strain at fracture (ε_f_), and modulus of elasticity (E). The tensile tests were conducted in a well-controlled ambient temperature.

## 3. Results and Discussion

### 3.1. The Influence of the Moisture Content on PLA Mechanical Properties

**Figure 5:**
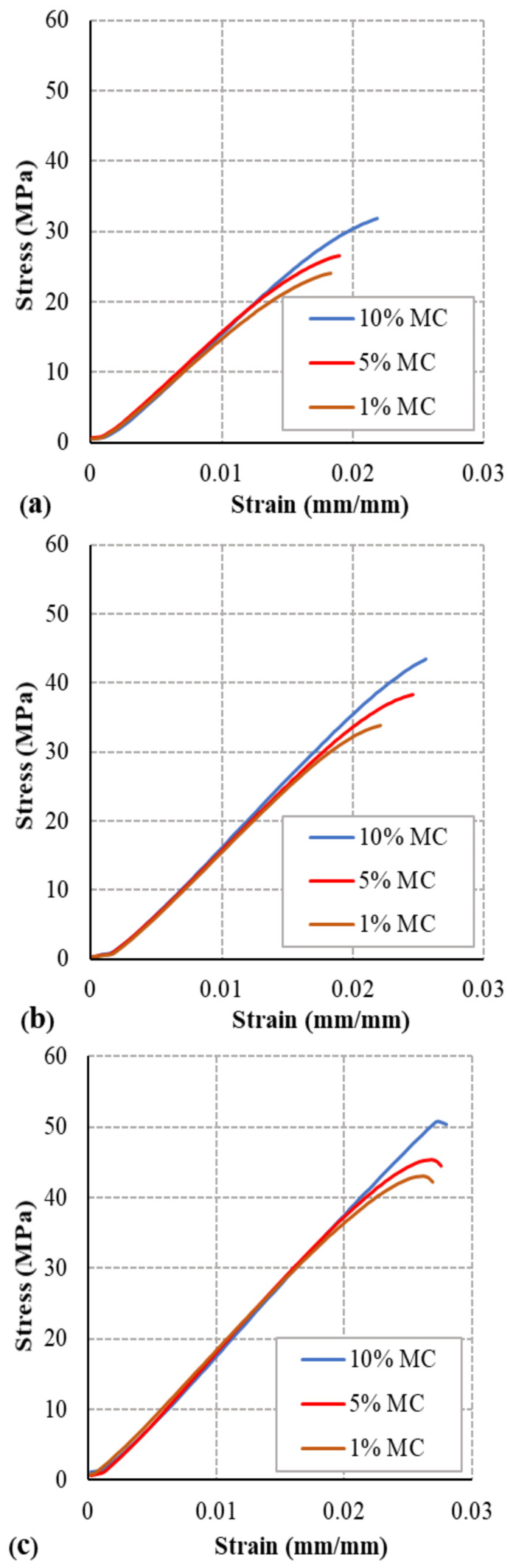
Tensile stress–strain curves for (**a**) 0° RA, (**b**) 45° RA, and (**c**) 90° RA.

The monotonic load tensile strength test of the 27 specimens was performed, and the mean values were plotted on a stress–strain graph. The curves show the tensile strength is affected by the moisture content percentage, whereas Young’s modulus (E) and strain at fracture (ε_f_) were less affected by the raster angle (Figure 5). The axial stress load behavior of the 0° RA specimens is shown in Figure 5a, where the highest strength is for the moistest specimen (10% moisture content) and strength decreases as the moisture content percentage decreases (5% moisture content and 1% moisture content). The stress–strain curves in Figure 5b for the specimens with 45° RA show similar behavior of the moisture content effect, yet higher UTS due to the raster angle orientation. The specimens with 10% moisture content and 90° RA Figure 5c have the greatest strength. The moisture content effect on the mechanical properties of PLA filament acts differently on ULTEM9085 filament as in Ref. [27]. The study in Ref. [27] shows that the UTS and strain at fracture decrease as the moisture content increases. However, the experimental results of PLA filament specimens with more moisture content have higher UTS and strain at fracture.

### 3.2. The Influence of the Raster Angle on PLA Mechanical Properties

**Figure 6:**
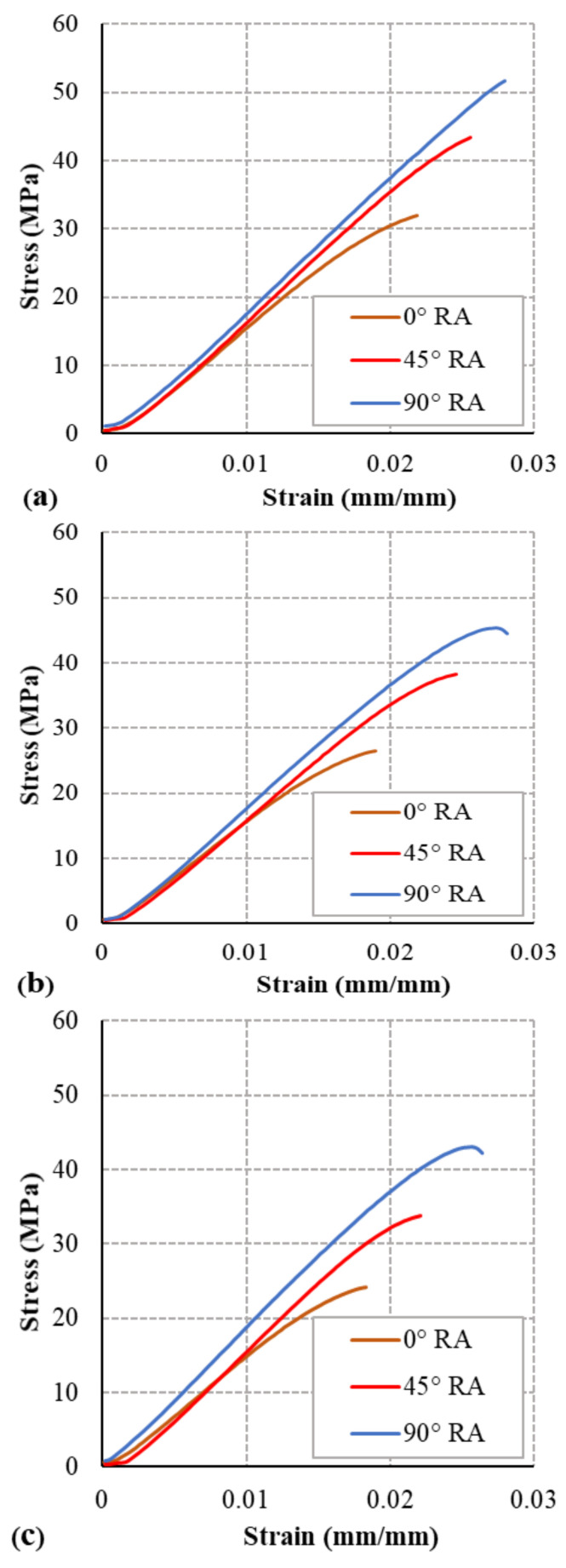
Tensile stress–strain curves for (**a**) 10% MC, (**b**) 5% MC, and (**c**) 1% MC.

The stress–strain curves show the tensile strength is significantly affected by the raster angle orientation. In contrast, the Young’s modulus (E) and strain at fracture (ε_f_) were less affected by the raster angle (Figure 6). The axial stress load behavior of the 0° infill angle specimens is shown in Figure 6a where the highest strength is for the specimen with 10% moisture content, and strength decreases as the moisture content percentage decreases. The stress–strain curves in Figure 6b for the specimens with 45° RA show similar behavior to the 0° RA specimens in Figure 6c, with higher UTS. The specimens with the 90° RA have the greatest strength, with a similar moisture content result as the other RA specimens. The performance of the PLA filament specimens with different raster angles acts in a similar way to ULTEM 9085 filament specimen’s performance presented in Ref. [12]. The UTS and strain at fracture increase as the raster angle is parallel to the loading direction.

### 3.3. Ultimate Tensile Strength

**Figure 7:**
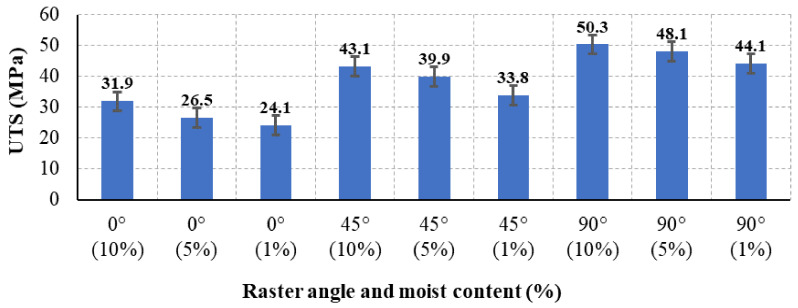
The ultimate tensile strength (UTS) for all specimens with different raster angles and moisture content.

The ultimate tensile strength of test specimens with various 3D printing parameters of raster angle and moisture content were analyzed. Figure 7 shows the results of different UTS for the 27 sets of specimens. The experimental results show that the UTS for the 0° RA is the lowest, and UTS increases as the raster angle changes from 45° to 90°. The UTS of the 90° RA increases by 36.5% when compared to the 0° RA. The weakness in the 0° RA is due to delamination of the layers during the loading process. Similarly, the results found in Refs. [14,25] shows the influence of the raster angle on the UTS of the PLA specimens increased by approximately 43%. The moisture content shows a significant effect on the UTS. The UTS for the 0° RA specimens decreases as the moisture content decreases. The results in Figure 7 show that the UTS decreased by 24.4% as the moisture content decreased from 10% to 1%. The variance of the UTS values is due to the loss of ductility as the moisture content decreases. Similarly, the effect of the moisture content shows the same effect in the other two raster angles, 45° and 90°. The UTS in the 45° RA decreased by 21.5%, whereas it decreased by 12.6% in the 90° RA. In summary, the highest UTS was obtained at 90° RA and 10% moisture content, whereas the lowest UTS was for the 0° RA and 1% moisture content. The decrease in moisture content decreases the UTS values.

### 3.4. Strain at Fracture

**Figure 8:**
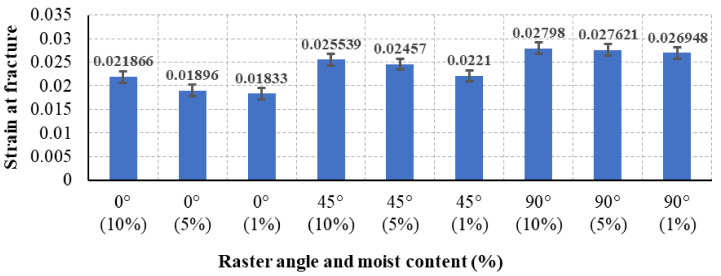
Strain at fracture in mm/mm for all specimens with different raster angles and moisture content.

The strain at fracture of test specimens with various 3D printing parameters of raster angle and moisture content are analyzed. The strain at fracture differs as the raster angle and moisture content change, as shown in the experimental results in Figure 8. The summarized data in Figure 8 show the significant effect of the raster angle on the strain at fracture. The experimental results show that the strain at fracture for the 0° RA is the lowest, whereas it increases as the raster angle increases from 45° to 90°. The strain at fracture of the 90° RA increases by 28% when compared to the 0° raster angle. Similar behavior was found in Refs. [14,25] where the influence of the raster angle on PLA specimens increased the strain at fracture by approximately 25%. However, the moisture content shows an insignificant effect in the strain at fracture. The strain at fracture for the 0° RA specimens slightly decreases as the moisture content decreases. The results in Figure 8 show that the strain at fracture decreased by 13.6% as the moisture content decreased from 10% to 1%. Similarly, the effect of the moisture content shows the same slight effect in the other two raster angles, 45° and 90°. The strain at fracture in the 45° RA decreased by 12.1% whereas it decreased by 3.6% in the 90° RA. In summary, the highest strain at fracture was reached at 90° RA and 10% moisture content, whereas the lowest strain at fracture was for the 0° RA and 1% moisture content.

### 3.5. Modulus of Elasticity

**Figure 9:**
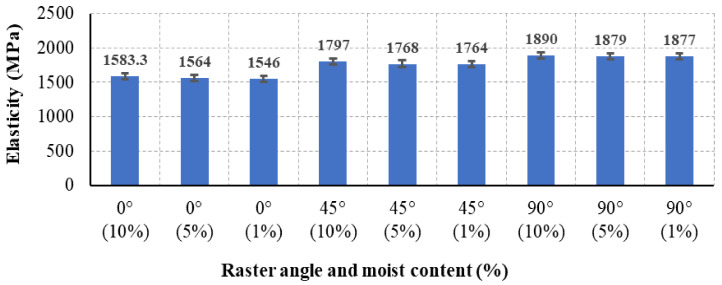
Elasticity in (MPa) for all specimens with different raster angles and moisture content (%).

The modulus of elasticity of test specimens with various 3D printing parameters of raster angle and moisture content are analyzed. The modulus of elasticity varies as the raster angle and moisture content changes as per the experimental data in Figure 9. The results in Figure 9 show a minor effect of the raster angle on the modulus of elasticity and a negligible effect of the moisture content on the modulus of elasticity. The lowest modulus of elasticity value is for the 0° RA, whereas it increases as the raster angle increases. Similar behavior of the influence of the raster angle on PLA specimens was found in Refs. [14,25] where the modulus of elasticity slightly increased (4%) as the raster angle increased. However, the moisture content has a negligible effect on the modulus of elasticity.

### 3.6. Fractured Specimens

**Figure 10:**
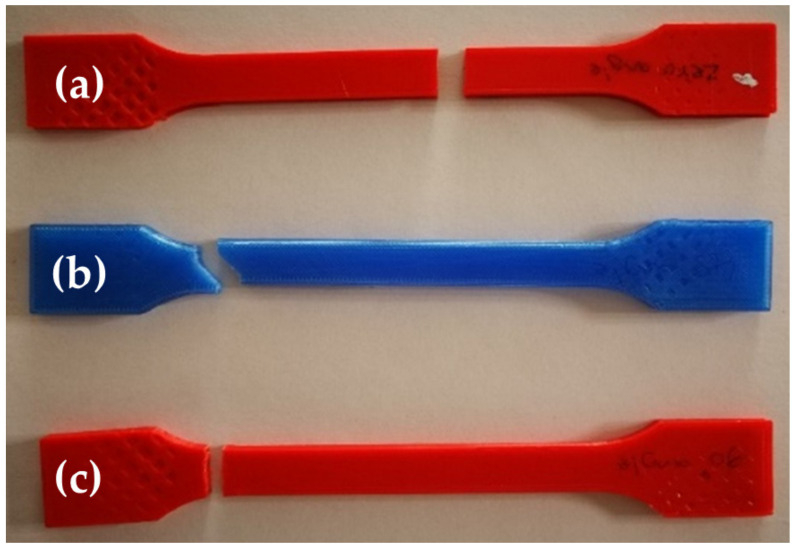
Specimens fractured due to tensile load. Specimens from top to bottom, (**a**) 0°, (**b**) 45°, and (**c**) 90°.

The fracture zones of the PLA 3D-printed specimens after tensile tests are shown in Figure 10. The specimens with 0° and 90° raster angles have flat fractured surfaces that are directed perpendicular to the loading direction. However, the fracture direction of the 45° RA specimens has a 45° slant surface. The fractured surface for the 0° and 45° RAs shows clearly that failure is due to the delamination of the layers while the 90° fractured surface shows failure due to filament breakage. The explanation of the variation in the fracture surface is the orientation of the built layers and the bonding between these layers during melting and cooling of the PLA filament, as shown in various studies [35,36,37,38]. Moreover, the fractured surface in all 27 specimens was relatively smooth, and no necking was observed. This fracture behavior is due to the brittleness property of pure PLA filament. The crack initiation and propagation for some specimens occurred in its middle part and for other specimens occurred in its end part. The differences in the crack initiation location are due to the FDM printing flaws which are caused by many factors such as machine vibration, uneven infill density, overheating, under-extrusion, or air gaps. Specimens with printing flaws are prone to high-stress concentration causing cracks to initiate and propagate.

## 4. Conclusions

Recent years have shown growth in research into three-dimensional printing techniques for additive manufacturing, investigating different fabrication techniques and parameters and their effect on the microstructure and mechanical properties of the printed objects. Predicting the mechanical behavior and performance of 3D-printed parts and components is vital in designing and engineering parts. In this study, an experimental investigation using tensile tests was carried out to assess the effect of raster angle and moisture content process parameters on the mechanical properties of PLA printed specimens. The conclusion of the research is as follows:

1. The raster angle and moisture content have a significant effect on the UTS. The UTS increased by 36% by reorienting the raster angle from 0° to 90°. In addition, the UTS decreased by 24.4% as the moisture content decreased from 10% to 1%.
2. The raster angle has a considerable effect on the strain at fracture, while the moisture content has an insignificant effect on the strain at fracture. The strain at fracture increased by 28% by reorienting the raster angle from 0° to 90°.
3. The infill angle has a minor influence on the modulus of elasticity, while the moisture content has a negligible effect on the strain at fracture. The modulus of elasticity increased by 18% by reorienting the raster angle from 0° to 90°.
4. The fractured surface in all 27 specimens was relatively smooth, and no necking was observed.

## Figures and Tables

**Table 1:** Polylactic acid (PLA) material characteristics by the manufacturer [33].

Ultimate Tensile Stress	Density	Strain at Fracture	Elasticity
62.63 MPa	1.24 g/cm^3^	0.044 (mm/mm)	2504 MPa

**Table 2:** Process factors and levels.

Control Factors	Level		
A: Raster Angle	0°	45°	90°
B: Moisture Content	1%	5%	10%

**Table 3:** Combination of parameters.

Experiment No.	Factors	Level
1	0°	1%
2	0°	5%
3	0°	10%
4	45°	1%
5	45°	5%
6	45°	10%
7	90°	1%
8	90°	5%
9	90°	10%

**Table 4:** Fixed printing parameters.

Nozzle diameter	0.4 mm	Deposition line width	0.48 mm
Nozzle extrusion temp.	240 °C	Deposition layer thickness	0.1 mm
Heated bed temp.	60 °C	Printing speed	30 mm/s
Infill density	100%	Fill gaps between walls	Everywhere

## Data Availability

The data presented in this study are available on request from the corresponding author.
